# Behçet disease – although rare can be present in children

**DOI:** 10.1186/1546-0096-12-S1-P353

**Published:** 2014-09-17

**Authors:** Aurelia Stancu

**Affiliations:** 1 Pediatrics, Emergency Childrens Hospital M.S. Curie, Bucharest, Bucharest, Romania

## Introduction

The child's history reveals frequent faringitis accompanied by oral lesions (4-5 episodes/year), billatreal ankle joint swelling and weight loss. Behçet disease (BD) is characterized by a triple-symptom complex of recurrent oral aphthous ulcers, genital ulcers, and uveitis. Hippocrates may have described Behçet disease in the fifth century BC; however, the first description of the syndrome was attributed to the Turkish dermatologist Hulusi Behçet in 1924. In 1930, the Greek physician Adamantiades reported a patient with inflammatory arthritis, oral and genital ulcers, phlebitis, and iritis. Since then, the syndrome has been referred to as Behçet disease.

## Objectives

Behçet disease is most common among persons aged 20-40 years. Cases that develop before age 25 years are more likely to involve eye disease and active clinical disease.The mean age at onset is 25-30 years.

## Methods

The author present the case of a 16 years adolescent that comes to our office for oral aphthous ulcers (for the last 3 months), genital ulcers and nodular lesions on the legs (for the last month).

## Results

The International Study Group for Behçet's Disease has emphasized the presence of recurrent oral ulcers as a primary consideration in the diagnosis of Behçet disease.In response, the pathogens above have been targeted for study in hopes of establishing a direct link between their presence and disease activity. Unfortunately, researchers have been unable to generalize results across geographic populations so far.

## Conclusion

Behçet disease, although rare, is present in children. This is why pediatricians with rheumatology concerns and beyond, need to know about it and to take it into consideration when needet. Clinical examniation of a child with symptoms similar to that we present, makes the diagnosis difficult.

## Disclosure of interest

None declared.

